# Readiness of the Kenyan public health sector to provide pre‐referral care for severe paediatric malaria

**DOI:** 10.1111/tmi.13728

**Published:** 2022-02-06

**Authors:** Beatrice Amboko, Beatrice Machini, George Githuka, Philip Bejon, Dejan Zurovac, Robert W. Snow

**Affiliations:** ^1^ KEMRI‐Wellcome Trust Research Programme Nairobi Kenya; ^2^ Division of National Malaria Programme Ministry of Health Nairobi Kenya; ^3^ Centre for Tropical Medicine and Global Health University of Oxford Oxford UK

**Keywords:** artesunate, Kenya, pre‐referral treatment, readiness, Severe malaria

## Abstract

**Objective:**

To assess readiness among primary public health facilities in Kenya to provide pre‐referral antimalarials for severe malaria.

**Methods:**

Nine national surveys of randomly selected primary public health facilities undertaken bi‐annually between 2017 and 2021 were analysed. The outcomes included the availability of pre‐referral antimalarial drugs at the health facilities and health worker knowledge of recommended pre‐referral treatment for severe malaria.

**Results:**

A total of 1540 health workers from 1355 health facilities were interviewed. Injectable artesunate was available at 46%, injectable quinine at 7%, and artemether at 3% of the health facilities. None of the facilities had rectal artesunate suppositories in stock. A total of 960 (62%) health workers were trained on the use of injectable artesunate. 73% of the health workers who had ever referred a child with severe malaria were aware that artesunate was the recommended treatment, 49% said that intramuscular injection was the preferred route of administration, and 60% stated the correct dose. The overall knowledge level of the treatment policy was low at 21% and only slightly higher among trained than untrained health workers (24% vs 14%; *p* < 0.001) and those with access to guidelines versus those without access (29% vs 17%; *p* < 0.001).

**Conclusions:**

The readiness of primary health facilities and health workers to deliver appropriate pre‐referral care to children with complicated malaria in Kenya is inadequate. Further investments are required to ensure (a) availability of nationally recommended pre‐referral antimalarials; (b) appropriate training and supervision in their administration, and (c) monitoring of the entire referral process.

## INTRODUCTION

Malaria is a disease that can rapidly evolve into severe, life‐threatening complications requiring emergency care and parenteral treatment [1, 2]. The referral, and associated pre‐referral care, of patients with signs of severe malaria is critical to case‐management strategies globally [3, 4]. In sub‐Saharan Africa, delays and lack of supportive care during the referral process have been shown to increase mortality among hospitalised malaria patients [5, 6]. Malaria referral practices have been described as inadequate following record reviews of severe malaria cases admitted to hospitals in Zambia [5], Uganda [7, 8], Tanzania [9], Mali [10], and the Democratic Republic of Congo [11]. At the primary care level, where referrals are initiated, reviews of health centre or community‐health worker records and practices in Kenya [12], Burkina Faso [13] and Uganda [14] have also shown poor compliance with appropriate practice. Importantly, verbal autopsy, confidential enquiries of events leading to deaths attributed to malaria in Tanzania, Mali, and Uganda have all demonstrated delays and inadequacies in the referral process as contributing to fatal outcomes [15, 16]. These studies highlight the complexity of referral processes including missed diagnosis of severe signs, the importance of distance between primary and secondary service providers, and lack of appropriate resources at primary health facilities to manage referred patients.

Randomized controlled trials have demonstrated that artesunate is superior to quinine during intravenous (IV) inpatient care [17] and that pre‐referral artesunate is superior to placebo for pre‐referral care [18]. In 2012, WHO recommended the use of intramuscular (IM) artesunate for pre‐referral treatment of suspected severe malaria [4, 19]. The use of a single dose of IM artesunate for pre‐referral treatment of suspected severe malaria is recommended across all age groups [4, 18]. If IM artesunate is unavailable, the use of IM artemether or IM quinine in the absence of artemether is recommended [4, 19]. In children under the age of 6 years, the use of a single dose of 10 mg/kg body weight of rectal artesunate suppositories (RAS) in absence of IM artesunate is also recommended if referral time is expected to be more than 6 h [4, 19, 20].

The availability of pre‐referral treatments at primary health facilities is clearly an essential prerequisite to the implementation of referral care and should be routinely monitored. Given the multiple options recommended by the WHO, the preferred treatments administered by frontline health workers should also be monitored. In this article, pre‐referral malaria treatment availability and health worker knowledge are described at primary public health facilities in Kenya between 2017 and 2021.

## METHODS

### Kenyan national standard guidelines for pre‐referral management of severe malaria

In 2012, the malaria treatment guidelines were revised to recommend the use of parenteral artesunate for pre‐referral severe malaria treatment [21]. In 2016, the guidelines were revised again to reflect recommended pre‐referral treatment of suspected severe malaria at primary health facilities where intravenous severe malaria treatment is not possible, but injections are available to include a single dose of intramuscular artesunate 2.4 mg/kg for adults and 3.0 mg/kg for children <20 kg before referral to a higher‐level facility [22]. In accordance with WHO guidelines, if artesunate is not available, the use of artemether 3.2 mg/kg or quinine 20 mg/kg is recommended. In settings where intramuscular injections are unavailable or not possible, children below the age of 6 years should be treated with a single dose of RAS and be referred immediately to an appropriate facility for further care [22].

Since the adoption of the new severe malaria treatment policy using artesunate, various activities have been undertaken to support the translation of the policy into practice. They included the procurement and nationwide distribution of injectable artesunate in 2014. The revision of national standard treatment guidelines in 2014 and 2016 to reflect the new treatment policy and distribution to health workers together with artesunate job aides. One‐day training sessions of frontline health workers located in hospitals (levels 4, 5, and 6) on the use of injectable artesunate were undertaken in April and May 2014. The training module covered the recognition of severe malaria signs and symptoms, pre‐referral, and inpatient severe malaria management, including video presentations on artesunate use and practical demonstrations of artesunate dilution and administration. Trained health workers cascaded the training to other health workers within their respective counties. Since 2016, the severe malaria management training module has been part of the routine annual 3‐day case management trainings managed by the Division of National Malaria Programme (DNMP) [23]. Since 2018, pre‐referral recommendations using 3.0 mg/kg single dose IM artesunate for malaria have been included in the Integrated Management of Newborn and Childhood Illness guidelines, without reference to IM artemether, quinine or RAS [24].

### Data sources

The Kenyan DNMP has undertaken bi‐annual national, cross‐sectional health facility surveys since 2010 to monitor the implementation of the outpatient ‘test and treat’ policy described in detail elsewhere [25, 26, 27]. During each round of the facility surveys, a proportionate, stratified random sample of health facilities was selected from the national master health facility list taking into consideration the facility type, ownership, and administrative boundaries to ensure national representativeness. At each of the surveyed facilities, data collection methods included health facility assessments, interviews with health workers providing consultations at the outpatient departments and exit interviews with febrile patients using structured questionnaires. Each facility was assessed to determine the availability of pre‐referral medicines for severe malaria. All health workers who provided clinical consultations in the outpatient departments were interviewed. Information on their access to the revised 2016 guidelines, in‐service training, and knowledge about the pre‐referral severe malaria treatment policy using self‐administered, multiple‐choice questions were collected. The present study includes nine rounds of surveys undertaken nationwide between February 2017 and June 2021. One survey round in 2020 was missed due to the COVID 19 pandemic travel restrictions imposed in Kenya. Data quality was assured through 5 days of training of the field workers, double‐entry into a Microsoft Access database, and comparisons of data files using a verification program in Microsoft Access and referring to paper‐based questionnaires.

### Definition of outcomes

The health system readiness outcomes were measured at the health facility and health worker levels. The outcome at the health facility level included the availability of injectable antimalarials on the survey day. At the health worker level, the outcomes included exposure to training, guidelines, and their knowledge about the use of IM artesunate for pre‐referral management of suspected severe malaria in children among health workers who said they had ever referred a child with severe malaria. A composite outcome defined as their knowledge about the use of artesunate in children, the recommended route of administration as IM, and dosing of IM artesunate according to weight of the children <20 kg was also examined.

### Statistical analysis

Descriptive analysis measuring levels was performed at the health facility and health worker levels to assess the health system readiness to implement the pre‐referral severe malaria treatment policy. Analysis has been restricted to primary health facilities managed by the public sector. Hospitals were excluded as these constitute the secondary destinations for referral. Level estimates are calculated for each year the surveys were undertaken as proportions with corresponding 95% confidence intervals (CI). Overall differences in proportions were tested using chi‐square and differences over time using simple logistic regression with time (year) as the only independent variable. All analyses were conducted using Stata version 17 (StataCorp, College Station, TX, USA).

## RESULTS

A total of 1540 health workers (range across years 171–353) from dispensaries and health centres managed by the public sector were interviewed at 1,355 facilities (range 153–307 per year) (Table 1). The majority of health workers (82%) and health facilities (82%) were managed by the Ministry of Health. Of the health workers interviewed, 942 (62%) reported having received in‐service training or on‐job orientation on injectable artesunate, without any perceptible increase in the reported training rates between 2017 and 2021 (Table 1; *p* = 0.93). A third of the health workers had access to the revised 2016 malaria guidelines, and this increased across the years (*p* = 0.023). A total of 345 (22%) health workers reported that they had never referred a child with severe malaria (Table 1).

**TABLE 1:** Characteristics of interviewed health workers and availability of antimalarials by survey year

Year	2017 *n* (%) [95% CI]	2018 *n* (%) [95% CI]	2019 *n* (%) [95% CI]	2020 *n* (%) [95% CI]	2021 *n* (%) [95% CI]	Total *n* (%) [95% CI]
No. of health facilities	302	291	307	153	302	1355
No. of health workers	353	339	348	171	329	1540
Facility ownership						
FBO/NGO^a^	58	67	49	30	71	275
Government	295	272	299	141	258	1265
Access to 2016 malaria guidelines	90 (25.5) [20.8–30.8]	111 (32.7) [27.4–38.6]	136 (39.1) [33.7–44.8]	72 (42.1) [34.2–50.4]	104 (31.6) [26.6–37.1]	513 (33.3) [30.8–35.9]
Training on injectable artesunate	225 (63.7) [58.4–68.7]	207 (61.2) [55.7–66.5]	217 (62.4) [57.1–67.3]	103 (60.2) [52.5–67.5]	208 (63.4) [58.1–68.4]	960 (62.4) [59.9–64.9]
3‐day malaria case‐management course	127 (56.4)	90 (43.5)	131 (60.4)	55 (53.4)	113 (54.3)	516 (53.8)
1‐day severe malaria management course	27 (12.0)	25 (12.1)	39 (18.0)	19 (18.5)	37 (17.8)	147 (15.3)
ETAT training	6 (2.7)	9 (4.4)	9 (4.2)	6 (5.8)	17 (8.2)	47 (4.9)
IMCI training	10 (4.4)	31 (15.0)	41 (18.9)	23 (22.3)	53 (25.5)	158 (16.7)
On‐job training/orientation from cascade	88 (39.1)	121 (58.5)	121 (55.8)	58 (56.3)	134 (64.4)	522 (54.4)
Other^b^	8 (3.6)	18 (8.7)	17 (7.8)	6 (5.8)	25 (12.0)	74 (7.7)
No. of HWs never referred a child with severe malaria	80 (22.7) [18.5–27.4]	78 (23.0) [18.7–27.9]	91 (26.2) [21.7–31.2]	36 (21.1) [15.5–27.9]	60 (18.2) [14.4–22.8]	345 (22.4) [20.4–24.6]
Availability of antimalarials at the surveyed health facilities						
Artesunate (AS) injection	145 (48.0) [42.4–53.7]	125 (43.0) [37.4–48.7]	148 (48.2) [42.7–53.8]	73 (47.7) [39.9–55.6]	134 (44.4) [38.9–50.0]	625 (46.1) [43.4–48.8]
Artemether injection	14 (4.6) [2.8–7.7]	6 (2.1) [0.9–4.5]	5 (1.6) [0.7–3.9]	6 (3.9) [1.8–8.5]	13 (4.3) [2.5–7.3]	44 (3.3) [2.4–4.3]
Quinine injection	39 (13.0) [9.6–17.3]	22 (7.6) [5.1–11.3]	13 (4.3) [2.5–7.2]	3 (2.0) [0.6–5.9]	18 (6.0) [3.8–9.3]	95 (7.0) [5.8–8.5]
Any injectable antimalarial	163 (54.0) [48.3–59.5]	135 (46.4) [40.7–52.2]	156 (50.8) [45.2–56.4]	79 (51.6) [43.7–59.5]	154 (51.0) [45.4–56.6]	687 (50.7) [48.0–53.4]

^a^ FBO/NGO‐ faith based organization/non‐governmental organization.

^b^ Includes pre‐service training, self‐training, continuous medical education sessions, and guidelines/job aides.

Only 625 (46%) facilities had injectable artesunate in stock on the day of the survey, far fewer (44, 3%) had injectable artemether, while quinine was available in 95 (7%) facilities (Table 1). Overall, only 687 (51%) facilities had any injectable antimalarial for pre‐referral treatment available, and availability varied little between 2017 and 2021 (Table 1). None of the facilities had RAS in stock at any time between 2017 and 2021.

Among 1195 health workers who had reported ever referring a child with suspected or confirmed severe malaria and were asked about the recommended drug for pre‐referral management of suspected severe malaria, 73% stated artesunate, 4% artemether, and 12% quinine (Table 2). Among the health workers who said that artesunate was the recommended treatment for pre‐referral treatment of severe malaria, 427 (49%) knew that IM is the preferred route of administration followed by 373 (43%) reporting IV slow bolus. The correct dose of IM artesunate was correctly identified for children weighing ≤20 kg by 517 (60%) health workers (Table 2). Knowledge level about the treatment policy increased (66% to 78%, *p* < 0.001), IM as the preferred route decreased (59% to 44%, *p* = 0.001), and the dosing recommendations did not vary since 2017 (54% to 64%, *p* = 0.063) (Table 2).

**TABLE 2:** Pre‐referral artesunate treatment policy knowledge among health workers who have ever referred a child with severe malaria

Year	2017 *n* (%)	2018 *n* (%)	2019 *n* (%)	2020 *n* (%)	2021 *n* (%)	Total *n* (%)
*N*	271	261	257	135	268	1195^a^
The recommended pre‐referral treatment for children						
Artesunate (AS)	180 (66.4)	178 (68.2)	194 (75.5)	102 (75.6)	210 (78.2)	864 (72.5)
Artemether	12 (4.4)	11 (4.2)	9 (3.5)	3 (2.2)	11 (4.1)	46 (3.9)
Quinine	49 (18.1)	36 (13.8)	23 (9.0)	12 (8.9)	17 (6.3)	137 (11.5)
AL	23 (8.5)	31 (11.9)	27 (10.5)	17 (12.6)	26 (9.7)	124 (10.4)
DHA‐PPQ	2 (0.7)	2 (0.8)	1 (0.4)	0	0	5 (0.4)
Other	1 (0.4)	0	1 (0.4)	1 (0.7)	0	3^b^ (0.3)
Do not know	4 (1.5)	3 (1.2)	2 (0.8)	0	1 (1.5)	13 (1.1)
*N*	180	178	194	102	210	864
Preferred route of AS administration among those who stated AS as preferred drug						
Intramuscular	107 (59.4)	90 (50.6)	97 (50.0)	41 (40.2)	92 (43.8)	427 (49.4)
IV slow bolus	64 (35.6)	77 (43.3)	82 (42.3)	54 (52.9)	96 (45.7)	373 (43.2)
IV drip (infusion)	4 (2.2)	8 (4.5)	9 (4.6)	5 (4.9)	15 (7.1)	41 (4.8)
Intrarectal	3 (1.7)	0	2 (1.0)	1 (1.0)	3 (1.4)	9 (1.0)
Oral	0	0	0	0	1 (0.5)	1 (0.1)
Do not know	2 (1.1)	3 (1.7)	4 (2.1)	1 (1.0)	3 (1.4)	13 (1.5)
AS dosing in children <20 kg among those who stated AS as preferred drug						
3.0 mg/kg	96 (54.2)	111 (62.4)	108 (55.7)	68 (66.7)	134 (63.8)	517 (60.1)
2.0 mg/kg	10 (5.7)	9 (5.1)	16 (8.3)	6 (5.9)	16 (7.6)	57 (6.6)
2.4 mg/kg	47 (26.6)	35 (19.7)	46 (23.7)	14 (13.7)	41 (19.5)	183 (21.3)
3.6 mg/kg	2 (1.1)	2 (1.1)	2 (1.0)	0	2 (0.9)	8 (0.9)
4.2 mg/kg	0	1 (0.6)	1 (0.5)	1 (1.0)	0	3 (0.4)
5.0 mg/kg	4 (2.3)	1 (0.6)	0	1 (1.0)	1 (0.5)	7 (0.8)
Other	4 (2.3)	1 (0.6)	3 (1.6)	1 (1.0)	1 (0.5)	10^c^ (1.2)
Do not know	14 (7.9)	18 (10.1)	18 (9.3)	11 (10.8)	15 (7.1)	76 (8.8)

^a^ 3 missing values.

^b^ Does not give antimalarials or just manage symptoms.

^c^ 1.2 mg/kg or calculate based on the weight.

The overall knowledge of the pre‐referral treatment policy measured by the composite outcome of artesunate knowledge, route of administration, and dosing was low at 21% among health workers who had ever referred a child with severe malaria (Table 3). The overall knowledge levels were higher (24%) among health workers who had received training on the use of injectable artesunate than untrained health workers (14%) (*p* < 0.001). Similarly, the levels were higher in health workers who had access to the current malaria guidelines (29%) than those without access to the guidelines (17%) (Table 3; *p* < 0.001).

**TABLE 3:** Pre‐referral artesunate treatment policy, IM route of administration and dosing knowledge among health workers who have ever referred a child with severe malaria by training on injectable artesunate

Year	2017 *n*/*N* (%) [95% CI]	2018 *n* (%) [95% CI]	2019 *n* (%) [95% CI]	2020 *n* (%) [95% CI]	2021 *n* (%) [95% CI]	Total *n* (%) [95% CI]
Among those who had training on injectable artesunate	46/184 (25.0) [19.3–31.7]	38/180 (21.1) [15.8–27.6]	49/185 (26.5) [20.6–33.4]	17/89 (19.1) [12.2–28.7]	46/174 (26.4) [20.3–33.7]	196/812 (24.1) [21.3–27.2]
Among those who had no training on injectable artesunate	9/87 (10.3) [5.4–18.8]	11/81 (13.6) [7.3–23.9]	9/72 (12.5) [6.2–23.5]	7/46 (15.2) [7.7–28.0]	16/93 (17.2) [10.8–26.3]	52/379 (13.7) [10.5–17.7]
Among those who had access to guidelines	26/76 (34.2) [24.3–45.7]	27/97 (27.8) [19.8–37.6]	31/105 (29.5) [21.6–38.9]	9/57 (15.8) [8.5–27.4]	29/93 (31.2) [22.5–41.4]	122/428 (28.5) [24.4–33.0]
Among those without access to guidelines	29/195 (14.9) [10.5–20.7]	22/164 (13.4) [8.9–19.6]	27/152 (17.8) [12.2–25.2]	15/78 (19.2) [12.0–29.4]	33/175 (18.9) [13.7–25.4]	126/764 (16.5) [12.0–19.4]
Total	55/271 (20.3) [15.8–25.6]	49/261 (18.8) [14.4–24.1]	58/257 (22.6) [17.8–28.2]	24/135 (17.8) [12.3–25.0]	62/268 (23.1) [18.4–28.6]	248/1192 (20.8) [18.6–23.3]

## DISCUSSION

To effectively support pre‐referral treatment of severe paediatric malaria at primary health facilities, recommended drugs should be in stock. During the present study of 1355 primary public health facilities surveyed between 2017 and 2021, nearly half had none of the nationally recommended IM preparations available on the day of the survey. Despite a national guideline recommendation for RAS [22], this was absent from any of the surveyed facilities and none of the health workers interviewed suggested that this would be a pre‐referral treatment of choice. Kenya has not procured RAS for severe malaria case‐management, and it is absent from essential drug procurements across sub‐Saharan Africa [28], despite WHO recommendations. This might be due to lack of a quality assured RAS on the market that resulted in Medicines for Malaria Ventures (MMV) to collaborate with Cipla Limited and Strides Arcolab Limited with funding from UNITAID for the development and prequalification by the WHO of RAS in 2014 [29]. In 2018, Cipla limited together with MMV received approval from the WHO Prequalification Programme for their 100 mg RAS for the pre‐referral management of severe malaria [30]. In‐service training in the management of severe malaria had reached over 61% of the interviewed health workers, predominantly as part of the annual 3‐day broad malaria case‐management in‐service trainings and related cascaded on‐job training by trainers of trainers. However, only 88% of health workers who had referred a patient with severe malaria could correctly identify one of the parenteral, nationally recommended pre‐referral treatments and 60% could correctly identify the correct dose for children weighing ≤20 kg. Among health workers who had reported having received in‐service training in severe malaria, had seen a patient with severe malaria and correctly identified artesunate as the preferred drug, the correct knowledge about paediatric administration and dosing was 24% versus 14% among those who had not received any training. Training *per se* is not always the panacea to improved health worker performance [31],however, our results suggest that orientation of health workers to the correct policy recommendations for pre‐referral treatment impacts awareness. Anticipating that all health workers would remember dosing for drug preparations for rare disease events might be considered a high expectation. Most practitioners would refer to national formulary and standard treatment guidelines, while in our study, only a third of the interviewed health workers had access to the most up‐to‐date guidelines for severe malaria.

Significant improvements have been described in the management of uncomplicated malaria in Kenya since 2010, with decreasing stock‐outs of first line therapeutics and diagnostics and increasing testing rates and compliance with national standard treatment guidelines [25, 26, 27]. However, there remains a critical gap in the essential drug supply and in‐service training for pre‐referral malaria case‐management. This element of the case‐management pathway is often neglected and disconnected, with a focus on hospital care or primary care, without considering the referral pathway connecting both levels.

The referral process is multifaceted and requires more than simple pharmaceutical intervention, broader integrated clinical management training is required to improve detection of danger signs [32], Emergency Triage and Treatment (ETAT) [33, 34] and improved transport for emergency care facilities from the periphery [35]. The Kenyan Ministry of Health has begun to identify how this might be strengthened as part of revised referral guidelines [36]. However, notwithstanding the need for a comprehensive approach, making essential medicines to support pre‐referral care and in‐service training to support their appropriate use would be an important first step.

The survey did not include direct observations of practices related to the management of severe, complicated malaria. These events are rare, and observational studies of health workers in these situations has ethical concerns. Nor was the survey designed to investigate health workers knowledge of broader IMCI/ETAT danger signs and their pre‐referral treatments (anticonvulsants, antibiotics) or advise given to caregivers. Further studies on patients, health service providers, and facility readiness are required to specifically address referral practices, from community to primary care to hospital gates and ward admission. These studies would provide a greater depth of knowledge to support improvements in the pre‐referral care of patients with complicated malaria.

## CONCLUSION

The readiness of health facilities and health workers to deliver appropriate pre‐referral care to children with complicated malaria in Kenya remains inadequate. Further investments are required to ensure the availability of nationally recommended pre‐referral antimalarials, appropriate training, supervision, and mentorship in their administration and routine quality of care monitoring.
